# Design, Characterization and Pharmacokinetic–Pharmacodynamic Evaluation of Poloxamer and Kappa-Carrageenan-Based Dual-Responsive In Situ Gel of Nebivolol for Treatment of Open-Angle Glaucoma

**DOI:** 10.3390/pharmaceutics15020405

**Published:** 2023-01-25

**Authors:** Pradeep Singh Rawat, Punna Rao Ravi, Shahid Iqbal Mir, Mohammed Shareef Khan, Himanshu Kathuria, Prasanna Katnapally, Upendra Bhatnagar

**Affiliations:** 1 Department of Pharmacy, BITS-Pilani Hyderabad Campus, Hyderabad 500078, Telangana, India; 2 Nusmetics Pte Limited, E-Centre@Redhill, 3791 Jalan Bukit Merah, #05-27, Singapore 159471, Singapore; 3 Vimta Labs Limited, 142, Cherlapally Main Rd, IDA Phase II, Hyderabad 500051, Telangana, India

**Keywords:** ocular drug delivery, nebivolol, open-angle glaucoma, in situ gel, Box Behnken design, Poloxamer-407, kappa-carrageenan

## Abstract

This study developed a dual-responsive in situ gel of nebivolol (NEB), a selective β-adrenergic antagonist. The gel could achieve sustained concentrations in the aqueous humor to effectively treat glaucoma. The gel was prepared using a combination of poloxamers (Poloxamer-407 (P407) and Poloxamer-188 (P188)) and kappa-carrageenan (κCRG) as thermo-responsive and ion-sensitive polymers, respectively. Box–Behnken design (BBD) was used to optimize the effect of three critical formulation factors (concentration of P407, P188 and κCRG) on two critical response variables (sol-to-gel transition temperature of 33–35 °C and minimum solution state viscosity) of the in situ gel. A desirability function was employed to find the optimal concentrations of P407, P188 and κCRG that yielded a gel with the desired sol-to-gel transition temperature and solution state viscosity. An NEB-loaded gel was prepared using the optimized conditions and evaluated for in vitro drug release properties and ex vivo ocular irritation studies. Furthermore, ocular pharmacokinetic and pharmacodynamics studies were conducted in rabbits for the optimized formulation. The optimized NEB-loaded gel containing P407, P188 and κCRG had a sol-to-gel transition temperature of 34 °C and exhibited minimum viscosity (212 ± 2 cP at 25 °C). The optimized NEB-loaded gel sustained drug release with 86% drug release at the end of 24 h. The optimized formulation was well tolerated in the eye. Ocular pharmacokinetic studies revealed that the optimized in situ gel resulted in higher concentrations of NEB in aqueous humor compared to the NEB suspension. The aqueous humor C_max_ of the optimized in situ gel (35.14 ± 2.25 ng/mL) was 1.2 fold higher than that of the NEB suspension (28.2 ± 3.1 ng/mL), while the AUC_0–∞_ of the optimized in situ gel (381.8 ± 18.32 ng/mL*h) was 2 fold higher than that of the NEB suspension (194.9 ± 12.17 ng/mL*h). The systemic exposure of NEB was significantly reduced for the optimized in situ gel, with a 2.7-fold reduction in the plasma C_max_ and a 4.1-fold reduction in the plasma AUC_0–∞_ compared with the NEB suspension. The optimized gel produced a higher and sustained reduction in the intra-ocular pressure compared with the NEB suspension. The optimized gel was more effective in treating glaucoma than the NEB suspension due to its mucoadhesive properties, sustained drug release and reduced drug loss. Lower systemic exposure of the optimized gel indicates that the systemic side effects can be significantly reduced compared to the NEB suspension, particularly in the long-term management of glaucoma.

## 1. Introduction

Glaucoma is a cluster of chronic, progressive optic neuropathies that manifests as an increase in intra-ocular pressure leading to damage of the optic disc and eventually impairing vision [1]. Glaucoma is the second most common cause of blindness globally. The major risk factor for the onset of primary open-angle glaucoma (POAG) is elevated intraocular pressure (IOP) [2]. The main cause of elevated IOP in the anterior chamber of the eye is an ocular drainage system malfunction, which results in blockage and resistance to aqueous humor outflow [3]. Currently, β-blockers account for approximately 70% of all prescriptions for treatment of POAG. This is due to their effectiveness and mechanism of competing with catecholamines for β-2 adrenoreceptors in the ciliary epithelium and decreasing aqueous humor production [4]. However, the non-selective nature of first-generation β-blockers leads to systemic side effects raising concern for long-term management of POAG [5]. Though the second-generation β-1 cardio-selective agents like betaxolol offer a better systemic safety profile as compared to the existing first-generation β-blocking agents, they still suffer from some systemic side effects [6].

Nebivolol (NEB), a third-generation novel selective β-adrenolytic drug, has an unique mechanism of facilitating nitric oxide release that plays a role in the L-Arginine/NO/cGMP pathway, which provides neuroprotective properties while modulating aqueous humor drainage from the trabecular meshwork [7]. These dual mechanisms of lowering IOP as well as providing neuroprotection offer an advantage in the long-term management of POAG [8].

Ophthalmic drops (solutions/suspensions) are the most popular and convenient drug products available for the treatment of ocular diseases. Ophthalmic drops are easy to administer, non-invasive and produce high patient compliance, particularly in ocular diseases which require long-term, multi-dose administration of the drug. Ocular drug delivery is riddled with many challenges due to various physiological, anatomical and enzymatic barriers. These challenges become increasingly difficult as the target site for drug distribution and action moves from superficial layers (layers of cornea/conjunctiva) of the eye to the inner tissue of the eye (iris/ciliary body/vitreous humor). In the management of POAG using conventional ophthalmic drops, the ocular bioavailability for many drugs is very minimal (around 5–10%) while the systemic exposure is very high (50– 90%) [9,10]. 

An in situ gel is an ideal choice for ocular drug delivery because of its ability to undergo rapid sol-to-gel transition via temperature/pH/ion stimuli [11]. Due to its solution state, it is easy to administer while maintaining dose accuracy. Following administration, the in situ gel form a viscous gel which helps in retaining the drug at the surface of the cornea and provides intimate contact with the cornea. In addition, drug dilution by lachrymal fluids and drug loss due to naso-lachrymal drainage is significantly reduced by the viscous gel. This results in higher ocular availability and lower systemic absorption of the drug. 

Several synthetic and natural polymers were investigated for their in situ gelling properties based on various stimuli such as temperature, ions and pH [12,13,14]. In glaucoma, there is dysfunction in aqueous humor circulation leading to variation in the pH and temperature at the precorneal area [15,16]. Therefore, it is crucial to design an ocular in situ gel that can be triggered by more than one stimuli as well as provide mucoadhesive characteristics to sustain drug release for effective management of glaucoma [17].

Poloxamer-407 (P407) is a synthetic thermo-responsive polymer. It is a triblock copolymer, consisting of a central hydrophobic polyoxypropylene (PPO) chain and two lateral hydrophilic polyoxyethylene (PEO) chains. P407 has 70% of PEO and 30% of PPO in its structure [18]. It exhibits thermo-responsive characteristics in the concertation range of 18–22% *w/v*. The temperature-induced gelation of P407 is due to the hydrophobic interaction of its copolymer chains. As temperature increases, the copolymer chains start aggregating to form a micellar structure, which is the initial step of gelation [19]. However, the gels produced are of low viscosity. Poloxamer-188 (P188) is added to increase the viscosity of the gels formed by P407. P188 has 80% of PEO and 20% of PPO in its structure. P188 produces viscous gels with good mucoadhesive characteristics even at lower concentrations. Therefore, most of the thermo-responsive in situ gels use a combination of P407 and P188 [20].

**Figure 1:**
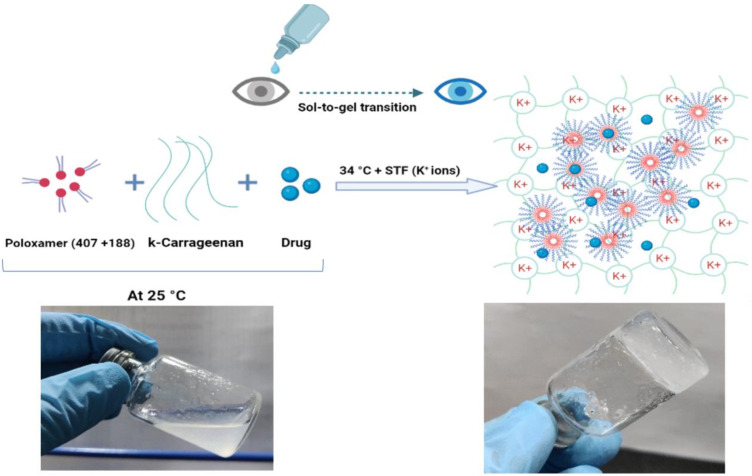
Illustration showing the possible gel matrix formed by the NEB-loaded dual-responsive in situ gel at 34 °C in presence of STF (containing K^+^ ions).

Carrageenan (CRG) is a long, linear polysaccharide containing D-galactose and D-anhydro-galactose disaccharide repeating units with anionic sulphate groups. Three different grades of CRG are available depending on the number of sulphate groups attached to the disaccharide repeating units. In kappa-carrageenan (κCRG), there is only one sulphate group attached to the disaccharide repeating units. The aqueous solutions of κCRG exhibit ion-sensitive in situ gelling properties in the presence of monovalent ions (such as Na^+^ and K^+^) [21,22]. κCRG can be combined with P407 + P188 to design dual-responsive ocular in situ gels for effectively delivering drugs towards the inner tissues of the eye. In addition, κCRG, when combined with P407 + P188, forms strong hydrogen bonds with their micellar structure and reinforces the gel structure (Figure 1), resulting in higher gel strength, improved mucoadhesion and slower gel erosion [23,24].

In the current research, we designed and optimized an NEB-loaded dual-responsive in situ gel using a mixture of P407 + P188 (as thermo-responsive polymer) and κCRG (as ion-sensitive polymer). The optimized formulation was characterized for mucoadhesion, in vitro drug release and ex vivo ocular toxicity. The ocular pharmacokinetic and pharmacodynamic studies were conducted in New Zealand white rabbits to determine the efficacy of the optimized dual-responsive in situ gel in comparison to the NEB suspension.

## 2. Materials and Methods

### 2.1. Materials

Nebivolol (NEB) and nebivolol-d4 (internal standard) were purchased from Vivan life sciences Private limited (Mumbai, India) and BioOrganics Private limited (Bangalore, India), respectively. κCRG, P407, P188 and benzododecinium bromide were procured from Sigma-Aldrich Private Limited (Mumbai, India). Methanol and acetonitrile (LC-MS grade) were purchased from Thermo Fischer Scientific (Mumbai, India). Ammonium acetate, formic acid and disodium EDTA were purchased from SRL Chem Limited (Mumbai, India). Sample analysis was conducted using high-quality HPLC-grade water obtained from the Milli-Q purification system (Millipore^®^, Burlington, MA, USA). Male New Zealand white rabbits (2–2.5 kg) were procured from Vimta Labs (Hyderabad, India).

### 2.2. Analytical Method for Analysis of NEB

A validated LC-MS/MS method, reported previously by our group, was used to analyse the concentration of NEB in the aqueous humor and plasma samples obtained in the ocular pharmacokinetic studies [25]. The samples were analysed using an Agilent HPLC (model: 1260 Infinity II, Agilent Technologies Inc., Santa Clara, CA, USA) coupled with a triple quadrupole mass analyser (model: API 4500, AB SCIEX, Redwood City, CA, USA). Chromatographic separation was performed on a reverse phase column (Zorbax SB-C18, 4.6 × 100 mm, 3.5 μm) using a mobile phase consisting of an organic phase (mixture of methanol and acetonitrile in the ratio of 70:30 *v/v*) and an aqueous phase (5 mM ammonium acetate buffer adjusted to pH 3.5 ± 0.05 with formic acid) in the ratio of 75:25 *v/v*. Samples were extracted using a protein precipitation technique. The mass spectrometer was operated in positive electrospray ionization mode with multiple reactions monitoring for NEB at (Q1→Q3) of (406.2→151.1) and for nebivolol-d4 (internal standard) at (Q1→Q3) of (410.2→151.3).

A validated RP-HPLC method was used to determine the concentration of NEB obtained in drug content analysis samples and in vitro drug release study samples. An end-capped C18 column (Luna®, 150 mm × 4.6 mm, 5 μm, Phenomenex, Torrance, CA, USA), maintained at 30 °C inside a column oven, was used in the analysis. The mobile phase consisting of acetonitrile and 0.1% *v/v* orthophosphoric acid in the ratio of 43:57 *v/v* was pumped at a flow rate of 0.8 mL/min to separate the NEB from the interfering peaks. The analyte was detected at a wavelength of 281 nm. The injection volume was fixed at 50 µL. The baseline was stabilized prior to the analysis of the samples.

### 2.3. Preparation and Optimization of NEB-Loaded Dual-Responsive In Situ Gel

#### 2.3.1. Preparation of NEB-Loaded Dual-Responsive In Situ Gel

The poloxamer solutions were prepared by the cold method [9]. In the first step, the required amounts of P407 (18–20% *w/v*, varied as per the design in BBD) and P188 (1–5% *w/v*, varied as per the design in BBD) were added to pre-cooled (4 °C) deionized water with continuous stirring to ensure proper hydration of the poloxamers. The resultant mixture was kept in the refrigerator for 24 h until the polymers were completely dissolved. The required amount of κCRG (0.3–0.5% *w/v*, varied as per the design in BBD) was added to the above solution and stirred at 500 rpm for 2 h to form a homogenous solution. Finally, NEB (0.3% *w/v*), mannitol (5.2% *w/v*; this was used as an isotonicity-adjusting agent) and benzododecinium bromide (0.01% *w/v*) were added to the resulting solution and stirred for 30 min to form an NEB-loaded dual-responsive in situ gel. The formulation was stored at 4 °C until further use.

#### 2.3.2. Optimization of NEB-Loaded Dual-Responsive In Situ Gel

Box–Behnken design (BBD), a response surface method, was used to analyse and optimize the effect of concentrations of P407, P188 and κCRG on the gelling temperature and solution state viscosity of the dual-responsive in situ gels [26]. BBD was employed at three experimental levels to optimize the three formulation-related factors: $X_{1}$—concentration of P407 (18% to 20% *w/v*); $X_{2}$—concentration of κCRG (0.3% to 0.5% *w/v*) and $X_{3}$—concentration of P188 (1% to 5% *w/v*) as shown in (Table 1). The effect of the three formulation factors was studied on two critical response variables of the in situ gel: $Y_{1}$: gelling temperature (°C) and $Y_{2}$: solution state viscosity at 25 °C (cP).

Design Expert software (Version 13, Stat-Ease Inc., Minneapolis, MN, USA) was used to construct the BBD for optimization of the dual-response in situ gel. In the optimization trials, a 17-run BBD (including five centre point runs) was constructed for the three formulation factors ($X_{1}$, $X_{2}$ and $X_{3}$) studied at three levels (−1, 0 and +1) to assess main effects, interaction effects and quadratic effects on response variables ($Y_{1}\;$ and $Y_{2}$). In order to evaluate the reproducibility of the method used in the preparation of the in situ gel, five centre point runs were included. Optimization using BBD resulted in a quadratic equation that relates each of the response variables, separately, with the critical factors. The general form of the second-order quadratic equation for a response variable is as follows:

(1) $$Y=\beta_{{}^{\circ}}+\beta_{1}X_{1}+\beta_{2}X_{2}+\beta_{3}X_{3}+\beta_{12}X_{1}X_{2}+\beta_{23}X_{2}X_{3}+\beta_{13}X_{1}X_{3}+\beta_{11}X_{1}^{2}+\beta_{22}X_{2}^{2}+\beta_{33}X_{3}^{2}$$

where Y is the response/dependent variable; $X_{1}$, $X_{2}$ and $X_{3}$ are input/independent variables, $\beta_{{}^{\circ}}$ is the arithmetic mean response of the seventeen runs. $\beta_{i}$ and $\beta_{ij}\;\left(i,\;j=1-3\right)\;$ are the coefficients of individual linear and quadratic effects of the factors, respectively. 

### 2.4. Characterization of Blank and NEB-Loaded Dual-Responsive In Situ Gels 

#### 2.4.1. Determination of Gelling Temperature and Solution State Viscosity of the In Situ Gels

The ‘vial tilting’ method, reported in the literature, was used to determine the gelling temperature of the in situ gels [27]. A small tube containing 1 mL of the test formulation was put in a thermostatically controlled water bath. The water bath temperature was raised steadily from 20 °C to 40 °C at a rate of 1 °C/min. The tube was turned 90 degrees at each temperature level. The temperature at which no flow was observed upon tilting the tube was identified as the gelling temperature of the formulation [28].

The solution state viscosity of the in situ gels was measured using a viscometer (Brookfield DV-E, AMETEK, Wilmington, MA, USA) with CP 52 spindle at 10 rpm [29]. The test formulation (in situ gel) was placed in a beaker, and the viscosity was measured at 25 ± 0.5 °C. The experiment was performed in triplicate. 

#### 2.4.2. Physical Appearance, pH and Drug Content of Optimized NEB-Loaded Dual-Responsive In Situ Gel

The physical appearance and clarity of the optimized NEB-loaded in situ gel was examined by visual observation. The pH of the formulation (for three replicates) was determined using a calibrated pH meter (Eutech Instruments, Pune, India) [30]. The drug content of the drug-loaded in situ gels was carried out by diluting 100 μL of the formulation in 1 mL of deionized water. The samples were then analysed using HPLC [31]. The drug content was determined for three replicate formulations.

### 2.5. Rheological Study of Blank and NEB-Loaded Dual-Responsive In Situ Gels

The rheological properties of the blank and NEB-loaded dual-responsive in situ gels were evaluated using a Rheometer (Anton Paar, MCR 302, Graz, Austria) to determine the sol-to-gel transition temperature and the strength of the gel formed by the formulations. The measurements were performed in oscillatory mode using parallel plate geometry with a temperature sweep from 20 °C to 37 °C. The samples were analysed in their linear viscoelastic regions, which were determined by the amplitude and frequency sweep experiments. Three different experimental conditions were used to evaluate the rheological behaviour of the samples: (1) temperature ramp, (2) temperature ramp in the presence of simulated tear fluid (STF) and (3) temperature ramp in the presence of deionized water. The sol–gel transition and gel strength were determined from the data obtained from the plots of ‘loss factor (tan δ) vs. temperature’ and ‘storage modulus (G’) vs. temperature’ [32,33,34,35].

### 2.6. Mucoadhesion Studies of NEB-Loaded In Situ Gels

The mucoadhesive property of the in situ gels was studied using a Texture analyser (TA-XT plus, Stable Micro Systems, Surrey, UK). A blank P407 + P188 in situ gel and blank κCRG in situ gel were prepared to understand the contribution of the thermo-responsive polymer (mixture of P407 + P188) and ion-responsive polymer (κCRG) towards the overall mucoadhesive properties of the dual-responsive in situ gel. In the study, a filter paper (Whatman filter paper, grade one, Size 110) was cut into a small disc and moistened with mucin dispersion (8% *w*/*w*, prepared in STF) to form a mucin disc (which can mimic the mucosal surface of the cornea). The mucin disc was then placed horizontally on the lower end of the texture profile analysis probe using double-sided adhesive tape. Around 100 µL of the test formulation (in situ gel) was poured near the basement probe where the temperature was maintained at 34 °C. The samples were equilibrated and allowed to undergo a sol–gel transition. The probe was lowered at a speed of 1 mm/s until the mucin disc came into contact with the surface of the gel formed by the test formulation. A downward force (0.2 N) was applied for 1 min to ensure proper contact between the mucin disc and the gel. The probe was then moved upwards at a speed of 0.5 mm/s. The force required to detach the mucin disc from the surface of the gel was determined from the force vs. time plot constructed by the instrument software. The study was conducted in triplicate [36,37].

### 2.7. In Vitro Drug Release Studies of NEB-Loaded In Situ Gels

The dialysis method was used to perform in vitro drug release studies of NEB-loaded in situ gels [30,38]. Drug release studies were conducted for the NEB suspension, NEB-loaded P407 + P188 in situ gel, NEB-loaded κCRG in situ gel and NEB-loaded dual-responsive in situ gel. In the study, 40 μL of the test formulation (equivalent to 3 mg of NEB per mL of formulation) was sealed in a dialysis bag (MWCO: 3.5 kDa). The dialysis bag was incubated in a beaker containing 100 mL of STF (pH 7.4 ± 0.5) with 0.5% *w/v* Tween 80 as the dissolution media. The dissolution media was stirred at 75 rpm while maintaining the temperature at 34 ± 0.5 °C. Samples of 2 mL were drawn at 0.5, 1, 2, 4, 6, 8, 12, 16, 18 and 24 h during the study. Fresh media (maintained at the same temperature) of equal volume was added each time the sample was drawn from beaker. The samples were centrifuged at 10,000 rpm. The supernatant was collected and analysed, after appropriate dilution, using HPLC to determine the concentration of NEB. The data obtained from the in vitro drug release studies were fit into various kinetic models (i.e., zero-order, first-order, Higuchi and Korsmeyer–Peppas models) to understand the release behaviour of NEB from the different in situ gels [39,40].

### 2.8. Ex Vivo Ocular Irritation Test (HET-CAM) of the Optimized In Situ Gels

The hen’s egg test on chorioallantoic membrane (HET-CAM) method, an inexpensive, rapid and sensitive alternative of the Draize test was performed to study the ocular irritation of optimized in situ gels. Eggs procured from a local hatchery were incubated for nine days for proper growth of the CAM. The eggshells were delicately cracked on each of eggs on the 10th day from the large end to expose the air cell without damaging the inner membrane. The inner membrane was carefully removed with forceps to make the CAM ready for studying the effect of four different treatments. Three eggs (*n* = 3) were used for each treatment. Group 1 was treated with 0.1 N NaOH (positive control), Group 2 was treated with 0.9% *w/v* NaCl solution (negative control), Group 3 was treated with the blank dual-responsive in situ gel and Group 4 was treated with the NEB-loaded dual-responsive in situ gel. Blood vessels were examined for 300 s for signs of vascular lysis (disintegration of blood vessels), haemorrhage and coagulation. The irritation score (𝐼𝑆) value for each treatment was determined using Equation (2).

(2) $$IS=\left[\frac{\left(301-H\right)}{300}\times 5\right]+\left[\frac{\left(301-L\right)}{300}\times 7\right]+\left[\frac{\left(301-C\right)}{300}\times 9\right]$$

where 𝐻 is the time (in sec) taken to start haemorrhage reactions, 𝐿 is the time (in sec) taken to start vessel lysis and 𝐶 is the time (in sec) taken to start coagulation formation on the CAM [41,42].

The ocular irritation properties of the treatments were identified based on the IS values. A treatment is considered to be ‘non-irritating’ if the IS value is in the range of 0–0.9, ‘slightly irritating’ if the IS value is in the range of 1–4.9, ‘moderately irritating’ if the IS value is in the range of 5–9.9 and ‘strongly irritating’ if the IS value is in the range of 10–21 [43].

### 2.9. Hemolysis Study of the Optimized In Situ Gels

The haemolytic study was conducted to evaluate the isotonicity of the optimized in situ gel. Blood (2 mL) was drawn from the marginal ear vein of rabbits using a syringe into centrifuge tubes pre-treated with anticoagulant (4% *w/v* disodium EDTA solution). Red blood cells were separated using centrifugation at 3600 rpm for 15 min. To achieve a haematocrit of 2% (*v/v*), the cells were suspended in a required volume of physiological saline. Then the RBC suspension (1 mL) was mixed with the (1 mL) in situ gel (blank dual-responsive in situ gel or NEB-loaded dual-responsive in situ gel), and the mixture was incubated in water at 37 ± 0.5 °C for 1 h. In positive and negative controls groups, a RBC suspension (1 mL) was mixed with 1 mL of Triton X-100 and 1 mL of 0.9% *w/v* NaCl solution, respectively. For each treatment, the supernatant collected after centrifugation was measured for ultraviolet absorbance at 540 nm, and the absorbance values were substituted in Equation (3) to determine the haemolysis (%). Haemolysis studies of the treatment were carried out in triplicate.

(3) $$\mathrm{Haemolysis}\;(\%)=\frac{\left(A_{s}-A_{b}\right)}{\left(A_{c}-A_{b}\right)}\times 100$$

where $A_{c}$ is the absorbance value of the supernatant obtained by treating RBC suspension with Triton X-100, $A_{s}$ is the absorbance value of the supernatant obtained by treating RBC suspension with in situ gelling formulation and $A_{b}$ is the absorbance value of the supernatant obtained by treating RBC suspension with a 0.9% *w/v* NaCl solution [44,45].

### 2.10. Ocular Histopathology Studies of In Situ Gels

Ocular histopathology studies were performed to evaluate the effect of optimized in situ gels on the structural integrity of the corneal epithelium. Fresh goat eyeballs were procured from a local slaughterhouse. The cornea was excised from the goat eyeball. The excised cornea was washed and then incubated with each treatment, separately, for 4 h. The treatments used in the study were: STF (pH 7.4 ± 0.5) (negative control), 75% *v/v* isopropyl alcohol (positive control), blank dual-responsive in situ gel and NEB-loaded dual-responsive in situ gel. After the incubation period, the cornea was again washed and fixed in a 10% formalin solution for 24 h. After fixation, the cornea was subsequently dehydrated for 1.5 h using ethyl alcohol (at each concentration gradient of 30–50–70–90–100%). The cornea was then placed in xylene for 1.5 h and embedded in hot paraffin at 56 °C for 24 h. Paraffin blocks were solidified at room temperature. A rotary microtome (Leica Microsystems SM2400, England) was used to slice paraffin tissue blocks (3–4 µm thick). The sliced tissues were mounted on a glass slide and washed with xylene to remove the paraffin. The tissues were finally stained with haematoxylin and eosin (H-E stain). The stained tissues were observed for histopathological changes under a digital microscope (ZEISS, Axiocam 705 color, Oberkochen, Germany) at 20× magnification [46].

### 2.11. In Vivo Studies of the Optimized NEB-Loaded Dual-Responsive In Situ Gel 

#### 2.11.1. Ocular Pharmacokinetic Studies

Ocular pharmacokinetic studies of the optimized NEB-loaded dual-responsive in situ gel and NEB suspension were performed in male New Zealand white albino rabbits. Animals (*n* = 6 for each treatment group) weighing between 2.5 and 3.0 kg and having clinically normal eyes (free from signs of ocular abnormality) were used in the study. The protocols of conducting the in vivo studies were approved by the Institutional Animal Ethics Committee (IAEC) of Vimta labs, Hyderabad, India (Protocol No.: VLL/1122/NG/1099). All the animals were acclimatized to animal facility conditions (22 ± 1 °C room temperature, 55 ± 10% RH, and 12 h light–dark cycle) for one week prior to the study. A calibrated micropipette was used to instil 40 µL of the test formulation (NEB suspension/NEB dual-responsive in situ gel) in the cul-de-sac of each of the eyes in all the rabbits. Immediately following the dosing, the upper and lower eyelids were gently held closed for 10 s to maximize the contact between the cornea and the administered formulation. Aqueous humor samples were collected under mild anaesthesia using isoflurane (2% *v/v*). Aqueous humor samples (70 μL) were collected from the anterior part of eye by puncturing it with a 30 G sterile hypodermic needle via paracentesis. Blood samples (0.25 mL) were collected from the animals by ear vein puncture and transferred to Eppendorf tubes containing 200 mM K2EDTA (20 µL per mL of blood) as an anticoagulant [47]. Aqueous humor and plasma samples were collected in a sparse sampling manner at 0.5, 1, 2, 4, 8, 12 and 24 h after the formulation instillation. The samples (blood and aqueous humor) obtained from the ocular pharmacokinetic study were analysed using a validated LC-MS method reported by our group [25].

A non-compartmental analysis was used to calculate the pharmacokinetic parameters from the NEB concentration versus time data in each of the matrices [48]. The maximum NEB concentration in the rabbit aqueous humor and plasma (C_max_, ng/mL), the time to reach C_max_ (T_max_, h) and the mean residence time (MRT_0–∞,_ h) were determined. The area under the curve from 0 to 24 h (AUC _(0–24)_, ng × h/mL) was calculated using the trapezoidal method.

#### 2.11.2. Pharmacodynamic Studies 

In the pharmacodynamic study, the intra-ocular pressure (IOP) in the eye of rabbits was measured using a calibrated tonometer (TONO-PEN XL, Reichert, Germany) [49]. The efficacy of the optimized NEB-loaded dual-responsive in situ gel was compared with the NEB suspension by comparing the time course of percent reduction in IOP [∆IOP(%)] of the two formulations. In the study, six rabbits were allocated to the two formulations, with three rabbits for each formulation. The pre-dose IOP values were measured in both the eyes of each rabbit before instilling the formulations. The formulations (NEB-loaded dual-responsive in situ gel and NEB suspension) were instilled at a dosing volume of 40 µL into the lower cul-de-sac of each of the eyes of the rabbits in their group. The IOP was measured at 2, 6 and 12 h after the ocular administration of the formulations. Based on the data obtained in the study, the percentage reduction in *IOP* [∆IOP(%)] at different time points was calculated for both the treatments using the following equation:

(4) $$∆IOP\left(\%\right)=\frac{\left(IOP_{Pre}-IOP_{t}\right)}{IOP_{Pre}}\times 100$$

where $IOP_{Pre}$ is the intra-ocular pressure at pre-dose (just before administering the treatment) and $IOP_{t}$ is the intra-ocular pressure at time *t* following the administration of the treatment [48].

## 3. Results

### 3.1. Optimization of Dual-Responsive In Situ Gels Using BBD

A total of 17 independent runs (including 5 centre point runs) were constructed using BBD to examine three critical formulation factors on the two response variables. NEB-loaded dual-responsive in situ gels were prepared, in triplicate, for each run separately based on the composition of the run given by the BBD. The prepared in situ gels were evaluated to determine their gelling temperature ($Y_{1}$) and solution state viscosity at 25 °C ($Y_{2}$). The data obtained for each of the runs are presented in Table 2. 

#### 3.1.1. Effect of Critical Formulation Factors on the Gelling Temperature (*Y*_1_) of In Situ Gels

Regression analysis was used to model the gelling temperature ($Y_{1}$) as a function of the three critical formulation factors ($X_{1},\;X_{2}\;\mathrm{and}\;X_{3}$) for the NEB-loaded dual-responsive in situ gels obtained from the 17 runs generated by BBD. The quadratic equation, with statistically significant terms, relating the gelation temperature of the in situ gels and the three critical factors, in the coded form, is given below:

(5) $$Gelling\;temperature\;\left(Y_{1}\right)=40.80-1.00X_{1}+0.125X_{2}+5.88X_{3}+0.25X_{2}X_{3}+0.225X_{1}^{2}-1.02X_{3}^{2}$$

The statistical significance of the regression model and the various model terms was evaluated using analysis of variance (ANOVA). The ANOVA results of the regression model for gelling temperature are presented in Table 3. The F_cal_ value (214.12) of the model was statistically significant, with *P_cal_* < 0.0001. The regression coefficients, R^2^_adj_ (adjusted R^2^) and R^2^_press_ (predicted error sum of square R^2^) of the model were 0.9917 and 0.9819, respectively. High R^2^_adj_ and R^2^_press_ indicate that the regression equation obtained in the optimization can predict the gelling temperature ($Y_{1}$) values within a less than 2% deviation from the experimental/observed values. The lack of fit of the model was insignificant (F_cal_ value = 0.417 and *P_cal_* = 0.751). The lowest and highest gelling temperatures were 33 ± 0.5 °C and 47 ± 0.5 °C for the in situ gels prepared using the conditions given in the 11th and 12th experimental runs, respectively (Table 2).

#### 3.1.2. Effect of Critical Formulation Factors on the Solution State Viscosity ($Y_{2}$) of In Situ Gels

The quadratic equation relating the effect of the three critical formulation factors ($X_{1},\;X_{2}\;\mathrm{and}\;X_{3}$) on the solution state viscosity ($Y_{2}$) of the NEB-loaded dual-responsive in situ gels obtained from the 17 runs generated by BBD, in the coded form, is presented in Equation (6) given below:

(6) $$Solution\;state\;vicosity\;\left(Y_{2}\right)=207.2+0.88X_{1}+2.88X_{2}-5.25X_{3}+X_{2}X_{3}-1.23X_{1}^{2}+2.53X_{3}^{2}$$

The results obtained from the ANOVA of the regression equation for solution state viscosity ($Y_{2}$) suggest that the model was statistically significant (F_cal_ value = 46.22 and *P_cal_* < 0.0001) while the lack of fit was insignificant (F_cal_ value = 0.361 and *P_cal_* = 0.786) (Table 3). The regression equation for solution state viscosity ($Y_{2}$) appeared to have very high predictability as suggested by R^2^_adj_ (0.963) and R^2^_press_ (0.923) values, which are closer to 1. 

In the optimization design, the in situ gels prepared using the experimental conditions given in the 12th run exhibited minimum viscosity (179 ± 2.3 cP at 25 °C), while the formulation prepared using the 11th experimental run conditions had maximum viscosity (233 ± 4.1 cP at 25 °C) (Table 2).

**Figure 2:**
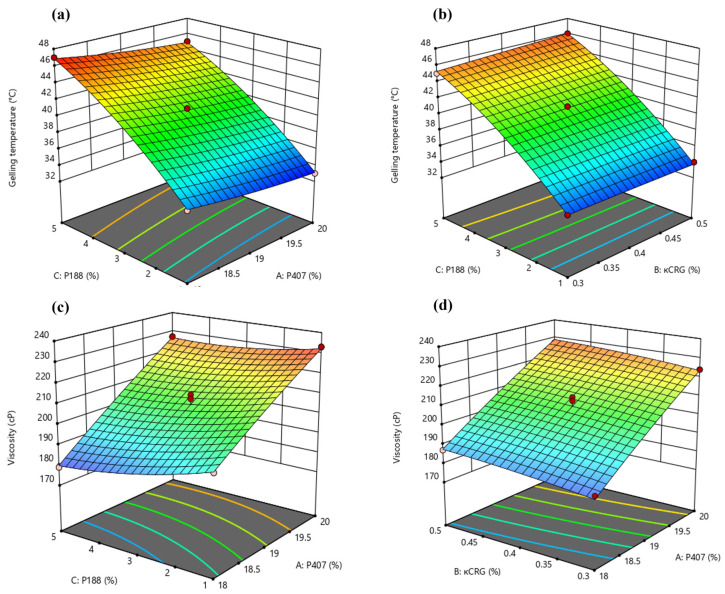
Response surface 3D plots showing the effect of (**a**) concentration of P407 and P188 on gelling temperature; (**b**) concentration of P188 and κCRG on gelling temperature; (**c**) concentration of P188 and P407 on solution state viscosity and (**d**) concentration of P407 and κCRG on solution state viscosity of NEB-loaded dual-responsive in situ gels.

The effect of concentration of P407 and concentration of P188, at a fixed concentration of κCRG, on gelling temperature of the in situ gels is presented as a response surface graph in Figure 2a. The gelling temperature decreased slightly with an increase in concentration of P407 (from 18 to 20% *w/v*) at higher concentrations of P188 (3 to 5% *w/v*). Increasing the concentration of P188 (from 1 to 5% *w/v*) had a positive impact on the gelling temperature of the in situ gels at any given concentration of P407 studied in the design. As depicted in Figure 2b, at a fixed concentration of P407, increase in the concentration of κCRG (from 0.3 to 0.5% *w/v*) did not have any significant effect on the gelling temperature of the in situ gels at any given concentration of P188 (1 to 5% *w/v*). It was expected κCRG, being an ion-sensitive polymer, should not have much impact on the gelling temperature of the in situ gels. Though P407 is a thermo-sensitive polymer, the concentration ranges in which it was studied had a smaller impact on gelling temperature. However, the gelling temperature increased with an increase in concentration of P188. This could be due to the increase in the polyethylene oxide content in the poloxamer polymers mixture (P407 + P188) in the vehicle, which then prevents the water molecules from moving away from PPO chains and thereby reduces the chances of micelle formation followed by gelling [50]. 

Figure 2c presents the response surface of solution state viscosity (at 25 °C) of the in situ gels as a function of the concentration of P188 and concentration of P407 (at a fixed concentration of κCRG). As depicted in the graph, the solution state viscosity decreased significantly with an increase in the concentration of P407 (from 18 to 20% *w/v*) at any given concentration of P188 (1 to 5% *w/v*). Increasing the concentration of P188 (from 1 to 5% *w/v*) marginally decreased the solution state viscosity of the in situ gels at any given concentration of P407 (18 to 20% *w/v*). At a fixed concentration of P188, an increase in the concentration of κCRG (from 0.3 to 0.5% *w/v*) resulted in a slight increase in the solution state viscosity of the in situ gels at any given concentration of P407 (18 to 20% *w/v*) (Figure 2d). The solution state viscosity of the in situ gels (at 25 °C) was more affected by the concentration of P407 than the concentration of P188 or κCRG. This could be due to the higher concentration of P407 relative to the other polymers used in the in situ gels. As the concentration of P407 increased, the solution state viscosity of the in situ gels (at 25 °C) increased. The results obtained in our study are consistent with the observations made by Hirun et al. in their work [26].

#### 3.1.3. Identification of Optimized Conditions Using Desirability Function

A simultaneous optimization technique involving a desirability function was employed to determine the optimal conditions for the preparation of NEB-loaded dual-responsive in situ gels. The objective for gelling temperature ($Y_{1}$) was set as a range between 33 and 35 °C, and for solution state viscosity, the goal was set to minimize while applying the desirability function. At the highest overall desirability value, the optimized conditions for the preparation of the NEB-loaded dual-responsive in situ gel were as follows: concentration of P407 = 19% *w/v*, concentration of κCRG = 0.3% *w/v* and concentration of P188 = 1% *w/v*.

### 3.2. Characterization of Optimized NEB-Loaded Dual-Responsive In Situ Gel

#### 3.2.1. Gelling Temperature and Solution State Viscosity

**Figure 3:**
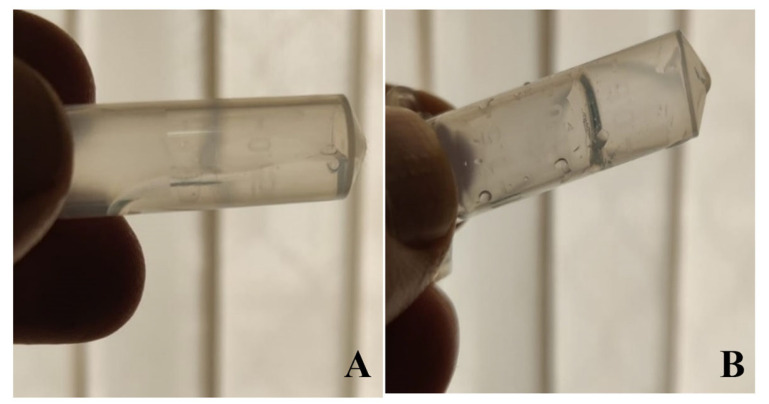
Image showing the flow properties of an optimized NEB-loaded dual-responsive in situ gel. (**A**) Free flowing properties at 25 °C suitable for easy and accurate dosing and (**B**) forming a firm gel at 34 ± 0.5 °C in presence of STF.

The gelling temperature and the solution state viscosity (25 °C) of the optimized NEB-loaded dual-responsive in situ gel were 34 ± 0.5 °C and 212 ± 2 cP, respectively. These results were close to the predicted values determined from the regression equations of gelling temperature ($Y_{1}$) and the solution state viscosity ($Y_{2}$), affirming the validity of the optimization model. The optimized NEB-loaded dual-responsive in situ gel exhibited desirable flow properties in the solution state (at 25 °C) while undergoing rapid sol-to-gel transition and forming a firm gel in the presence of STF at 34 ± 0.5 °C (Figure 3).

#### 3.2.2. Physical Appearance, pH, Osmolarity and Drug Content of the Optimized Dual-Responsive In Situ Gels

The blank dual-responsive in situ gel was transparent, while the optimized NEB-loaded dual-responsive in situ gel was translucent due to suspended NEB particles. The pH of both formulations was 7.2 ± 0.5, which is compatible with the pH of lachrymal fluids. The osmolarity of the optimized formulation was calculated based on the molarity equation and was found to be 285.44 mOsm/L. The osmolarity of the optimized in situ gel lies in the range reported for lachrymal fluids [51]. The drug content of the optimized NEB-loaded dual-responsive in situ gel was found to be 96.5 ± 1% for three independent batches of the formulation. This suggests that the method of preparation of the in situ gels was reliable and reproducible.

### 3.3. Rheological Studies of Blank and NEB-Loaded Dual-Responsive In Situ Gels

**Figure 4:**
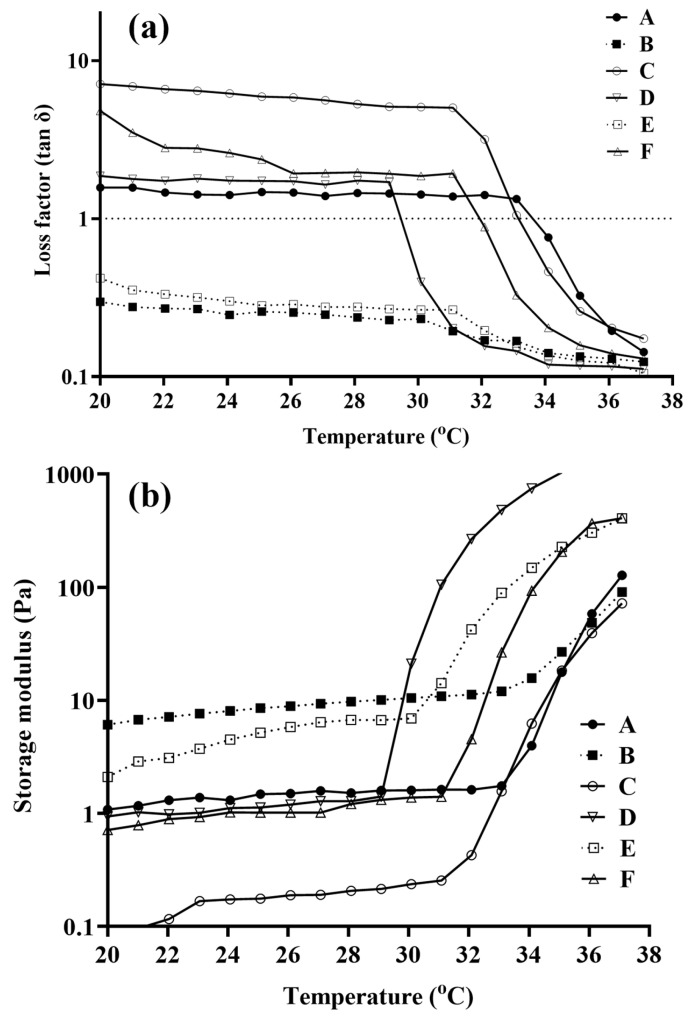
Semi-logarithmic plots of (**a**) loss tangent (tan δ) and (**b**) storage modulus (G’) of an optimized blank dual-responsive in situ gel and an NEB-loaded dual-responsive in situ gel as a function of temperature in presence of STF and deionized (DI) water. Rheological studies of optimized blank and NEB-loaded in situ gels. Note: A—blank dual-responsive in situ gel; B—blank dual-responsive in situ gel in the presence of STF; C—blank dual-responsive in situ gel in the presence of DI water; D—NEB-loaded dual-responsive in situ gel; E—NEB-loaded dual-responsive in situ gel in the presence of STF and F—NEB-loaded dual-responsive in situ gel in the presence of DI water.

Figure 4a,b depict the rheological behaviour of the optimized blank and NEB-loaded dual-responsive in situ gels, respectively, as a function of temperature in the presence of STF and deionized water. Figure 4a presents the loss factor (tan δ) vs. temperature behaviour of the formulations, while Figure 4b shows the storage modulus (G’) of the formulations as a function of temperature. The tan δ values of optimized blank and NEB-loaded dual-responsive in situ gels, without the addition of STF/deionised water, were more than one in the temperature range of 20 to 30 °C, indicating the free-flowing nature of the formulations. The tan δ values dropped below one between 32 and 34 °C for the optimized blank and NEB-loaded dual-responsive in situ gels in the presence of deionised water. This suggests a clear sol-to-gel transition due to the thermo-responsive component (P407 + P188) of dual-responsive in situ gels. In the presence of STF, tan δ values of the optimized blank and NEB-loaded dual-responsive in situ gels were more than one in the temperature range of 20 to 37 °C, with a drop in the range of 32 to 34 °C. Due to the presence of the cations (K^+^ and Na^+^) in STF, the ion-responsive component (κCRG) of the dual-responsive in situ gels caused the in situ gels to undergo sol-to-gel transition even at 20 °C. In the temperature range of 32 to 34 °C, the thermo-responsive component added to the increase in viscosity of the gel that was formed. These results indicate that both thermo-responsive and ion-responsive polymers were able to cause a sol-to-gel transition of the in situ gels independently and synergistically (Table 4).

The data obtained from the storage modulus (G’) of the in situ gels further supported the inferences made from the loss factor values. The G’ values of NEB-loaded dual-responsive in situ gels, in the presence of deionised water, were low in the temperature range of 20–31 °C. However, the G’ values increased steeply in the temperature range of 31 to 34 °C, suggesting a significant increase in the viscosity of the formulation due to the sol-to-gel transition caused by the thermo-responsive polymer. In the presence of STF, NEB-loaded dual-responsive in situ gels exhibited higher G’ values even in the temperature range of 20 to 30 °C, which further increased in temperature range of 30 to 34 °C. Higher G’ values even in the temperature range of 20 to 30 °C were due to the gelation of the ion-responsive polymer (κCRG) caused by the cations present in STF. The spike in G’ values in the temperature range of 30 to 34 °C was due to the increase in viscosity caused by the thermo-responsive polymer mixture (P407 + P188).

### 3.4. Mucoadhesion Study of the Blank In Situ Gels

**Figure 5:**
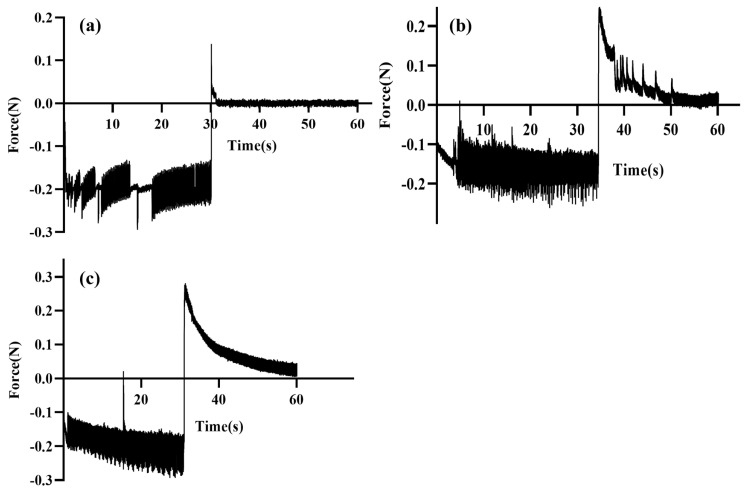
Mucoadhesive behaviour of (**a**) blank (P407 + P188) in situ gel, (**b**) blank κCRG in situ gel and (**c**) blank dual-responsive in situ gel, expressed in terms of mucoadhesion force (N).

In situ gels with good mucoadhesive characteristics can improve the overall permeation of the drug through the corneal membrane by providing intimate contact with the corneal membrane and also increasing the residence time. A texture analyser was used to evaluate the mucoadhesive properties of blank in situ gels. The blank P407 + P188 in situ gel (0.145 N) exhibited relatively low mucoadhesive properties compared to the bank κCRG in situ gel (0.253 N). This can be attributed to the large molecular weight and secondary interactions (hydrogen bonding) of κCRG with the mucin [52]. The blank dual-responsive in situ gel showed slightly more mucoadhesion compared to the blank κCRG in situ gel (0.289 N), possibly due to the additive effect of the individual polymers in the dual-responsive in situ gel (Figure 5).

### 3.5. In Vitro Drug Release Studies of NEB-Loaded In Situ Gels

**Figure 6:**
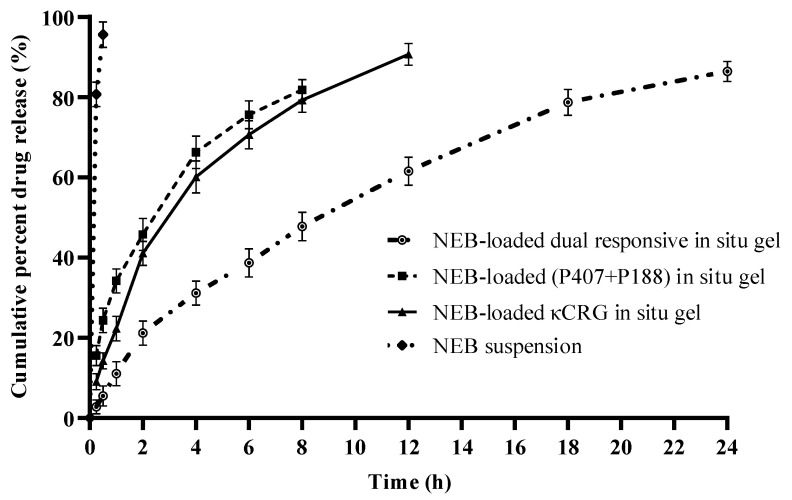
In vitro drug release profiles of an NEB suspension, NEB-loaded (P407 + P188) in situ gel, NEB-loaded κCRG in situ gel and NEB-loaded dual-responsive in situ gel. Each data point is the mean cumulative percent of NEB released (± SD) of three independent formulations (*n* = 3).

In vitro drug release studies were performed in STF (pH 7.4 ± 0.5) containing Tween 80 (0.5% *w/v*). The solubility of the NEB in STF was 28.62 µg/mL. Therefore, to maintain the sink condition, 100 mL of STF containing 0.5% *w/v* of Tween 80 was used in the study. The mean cumulative percentage of drug released vs. time was plotted from the in vitro dissolution data (Figure 6). The NEB suspension was dissolved completely within 30 min. The NEB-loaded P407 + P188 in situ gel and NEB-loaded κCRG in situ gel showed 90% drug release within 8 h and 12 h, respectively. The optimized NEB-loaded dual-responsive in situ gel slowed and prolonged the drug release, with 86% drug release at the end of 24 h. This can be attributed to the interaction of κCRG with the micelles of (P407 + P188) through secondary bonds, such as hydrogen bonds, resulting in increased viscosity of the gel formed, which is in line with the observations made from the rheological evaluation of the in situ gels. Increase in gel viscosity reduced the diffusivity of the drug through the gel matrix [23]. The drug release from the NEB-loaded dual-responsive in situ gel as well as the NEB-loaded P407 + P188 in situ gel and NEB-loaded κCRG in situ gel followed Higuchi kinetics. The value of n in the Korsmeyer–Peppas equation for the NEB-loaded dual-responsive in situ gel was found to be 0.77, suggesting the release of NEB was due to the combined effect of Fickian diffusion and matrix erosion.

### 3.6. Ex Vivo Ocular Irritation Test (HET-CAM) of the Optimized In Situ Gels

**Figure 7:**
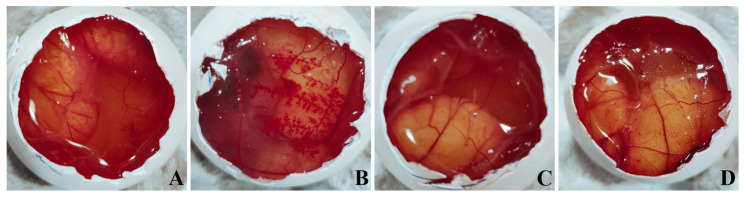
Images obtained from the HET-CAM test following the exposure of the CAM membrane to (**A**) negative control (0.9% *w/v* NaCl); (**B**) positive control (0.1 N NaOH); (**C**) blank dual-responsive in situ gel and (**D**) NEB-loaded dual-responsive in situ gel.

The images obtained from the HET-CAM test of the various treatments are presented in Figure 7. The positive control caused significant damage to the CAM within 30 sec, resulting in coagulation and haemorrhages followed by the lysis of blood vessels in the CAM (Figure 5B). The irritation severity score of the positive control was found to be 18. The negative control (0.9% *w/v* NaCl solution), blank and NEB-loaded dual-responsive in situ gels did not cause any inflammatory changes in the CAM. No visible changes were observed in terms coagulation/haemorrhage/lysis of the blood vessels in the CAM upon treatment with the negative control or in situ gels. The irritation severity score of the negative control, blank and NEB-loaded dual-responsive in situ gels were 0. Based on the results obtained from the HET-CAM test, it can be inferred that the optimized NEB-loaded dual-responsive in situ gel is safe and well tolerated by ocular tissues.

### 3.7. Hemolysis Study of the Optimized In Situ Gels

**Figure 8:**
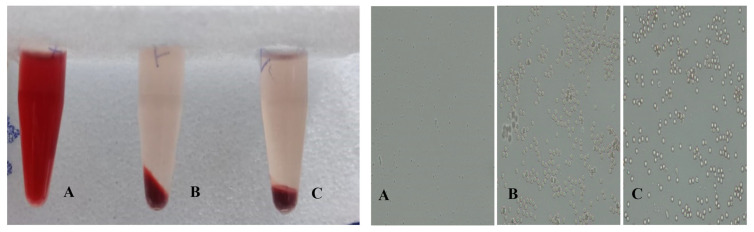
Results obtained from haemolysis studies of RBCs treated with (**A**) positive control (Triton X-100); (**B**) negative control (0.9% *w/v* NaCl) and (**C**) NEB-loaded dual-responsive in situ gel.

The RBCs treated with the optimized in situ gels (blank and NEB-loaded dual-responsive in situ gel) were checked for their shape and size (40× magnification). The morphology of the RBCs was found to be intact when treated with the negative control sample (STF pH 7.4), blank and NEB-loaded in situ gels. However, the RBCs incubated with Triton X-100 were completely lysed, as shown in Figure 8. The haemolysis (%) values of the RBCs incubated with the blank and NEB-loaded dual-responsive in situ gels were found to be 1.1% and 1.16%, respectively. These results suggest that optimized the NEB-loaded dual-responsive in situ gel is isotonic and biocompatible with no/minimal detectable disruption of RBCs.

### 3.8. Ocular Histopathology Studies of the Optimized In Situ Gels

**Figure 9:**
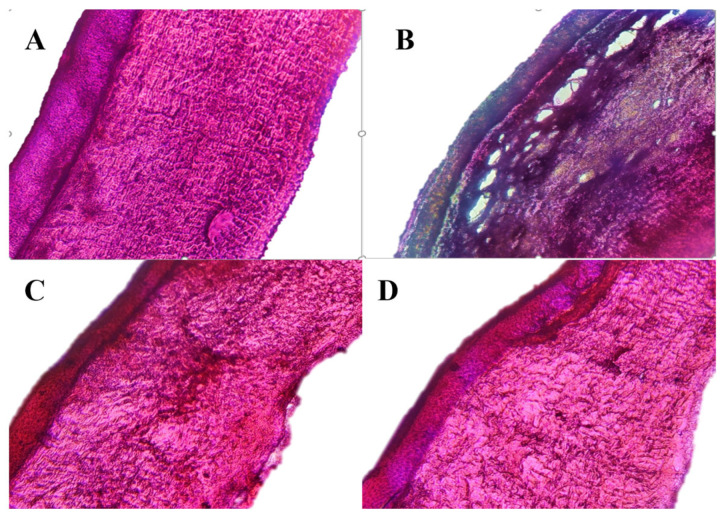
Microscopic images of the cornea exposed to (**A**) negative control (STF, pH 7.4); (**B**) positive control (IPA, 75% *v/v*); (**C**) blank dual-responsive in situ gel and (**D**) NEB-loaded dual-responsive in situ gel.

Microscopic examinations of the corneal structure incubated with STF (negative control) showed intact epithelium and stroma without any sign of tissue damage (Figure 9). There was visible disruption of the epithelium and stroma with tissue necrosis in the presence of 75% *v/v* isopropyl alcohol (positive control). The cornea treated with the optimized in situ gels (blank and NEB-loaded dual-responsive in situ gel) did not show any significant difference as compared to the STF-treated cornea. We can infer that the optimized formulations are safe and do not alter the structural integrity of the cornea.

### 3.9. In Vivo Studies of the Optimized NEB-Loaded Dual-Responsive In Situ Gel

#### 3.9.1. Pharmacokinetic Study

**Figure 10:**
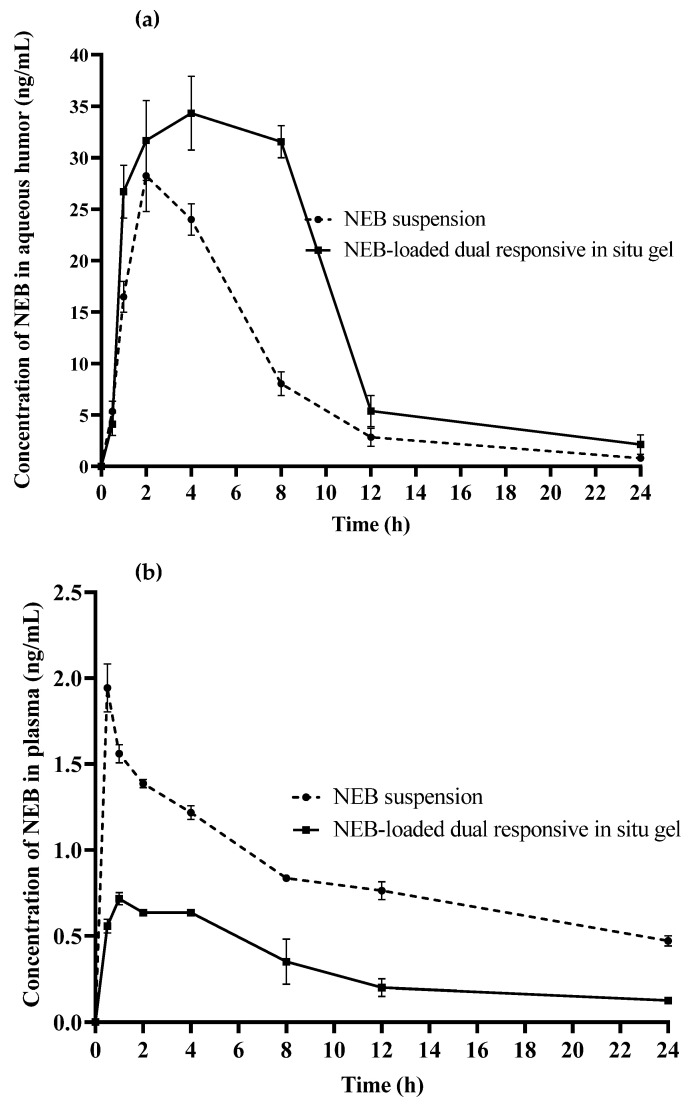
Mean concentration versus time profiles obtained following ocular administration of NEB suspension and optimized NEB in-situ gel in male New Zealand white rabbits (**a**) in aqueous humor and (**b**) in plasma. Each data point represents the mean of four independent determinations (*n* = 4).

The time course profiles of NEB in aqueous humor and plasma following the ocular administration of the NEB suspension and the optimized NEB-loaded dual-responsive in situ gel (at drug dose of 0.05 mg/kg) are shown in Figure 10a,b, respectively. The ocular and plasma pharmacokinetic data obtained in the study were subjected to non-compartmental analysis using Pheonix WinNonLin software (version 8.3.3.33, Pharsight Corporation, Raleigh, NC, USA) to determine pharmacokinetic parameters such as maximum concentration of NEB (C_max_), time to reach maximum concentration of NEB (T_max_), area under the course curve between zero to time ‘t’ (AUC_0-t_), area under the curve between ‘t = 0′ and ‘t = ∞’ (AUC_0–∞_) and mean residence time between ‘t = 0′ and ‘t = ∞’ (MRT_0–∞_). The pharmacokinetic parameters are presented in Table 5.

The aqueous humor C_max_ (35.14 ± 2.25 ng/mL) and AUC_0–∞_ (381.8 ± 18.32 ng/mL*h) of the NEB-loaded dual-responsive in situ gel were 1.2 fold (*P_cal_* < 0.05) and 2 fold (*P_cal_* < 0.0001) higher as compared with the C_max_ (28.2 ± 3.1 ng/mL) and AUC_0–∞_ (194.9 ± 12.17 ng/mL*h) of the NEB suspension, respectively. Higher C_max_ and AUC_0–∞_ suggests that a greater amount of NEB could permeate across the cornea and reach the aqueous humor in the case of the NEB-loaded dual-responsive in situ gel compared with the NEB suspension. This could be due to the lesser drug loss, lesser drug dilution and intimate contact between the gel and cornea for efficient permeation of the drug offered by the in situ gel compared to the suspension. Furthermore, the MRT_0–∞_ values of the in situ gel (8.11 ± 0.12 h) were significantly higher (*P_cal_* < 0.0001) than those of the NEB suspension (6.12 ± 0.18 h). This indicates that the in situ gel sustained the concentrations of NEB in the aqueous humor for a longer duration compared with the NEB suspension. This could be due to the ability of the in situ gel to resist nasolacrimal drainage for a longer duration compared to the suspension by forming a viscous gel at the precorneal area. Since the in situ gel remained in the precorneal area for a longer duration, the drug permeation into the aqueous humor was more sustained. 

In ocular drug delivery, systemic side effects resulting from unwanted absorption of the drug into systemic circulation is a major cause of concern. Ocular drug products of β-adrenergic antagonists (like timolol, betaxolol etc.) used in the long-term treatment of glaucoma suffer from systemic side effects like bradycardia, reduced blood pressure and an irregular pulse [53]. An ocular drug product which results in lesser systemic exposure of the drug will have a relatively low side effect profile. 

The C_max_ (0.69 ± 0.01 ng/mL) and AUC_0–∞_ (8.05 ± 0.43 ng/mL*h) in plasma of the NEB-loaded dual-responsive in situ gel were 2.7 fold (*P_cal_* < 0.0001) and 4.1 fold (*P_cal_* < 0.0001) lower as compared with the C_max_ (1.86 ± 0.01 ng/mL) and AUC_0–∞_ (33.21 ± 2.1 ng/mL*h) in plasma of the NEB suspension, respectively. Based on the data obtained, we can infer that the in situ gel resulted in significantly lower systemic exposure compared to the NEB suspension. Following ocular administration, the in situ gel forms a viscous gel layer with mucoadhesive properties on the surface of cornea through which the drug permeates into the aqueous humor. This pathway of drug permeation is considered more productive in reaching the target sites of the iris/ciliary body for the treatment of glaucoma. In the case of suspension, the drug present in a dissolved state in the lachrymal fluids could spread on the cornea and conjunctiva. Since the conjunctival membranes are highly vascularized, drugs that are in contact with conjunctiva permeate through it and reach systemic circulation. In addition, the naso-lachrimal drainage system can draw drugs present in the dissolved state into the lachrymal fluids and into the nasal cavity, from which the drug can get absorbed into systemic circulation. The plasma MRT_0–∞_ of the in situ gel (11.0 ± 0.6 h) was significantly (*P_cal_* < 0.0001) lower than that of the NEB suspension (25.8 ± 1.5 h). The concentrations of NEB in systemic circulation were sustained for more time in the case of the NEB suspension compared to the in situ gel. This suggests that the in situ gel significantly decreases the duration for which the systemic side effects would be experienced by the patients compared to the NEB suspension. Overall, the pharmacokinetic studies indicate that NEB-loaded dual-responsive in situ gels produce higher and sustained concentrations of NEB at the aqueous humor as well as reduce the intensity and duration of systemic side effects of the drug.

#### 3.9.2. Pharmacodynamic Study

**Figure 11:**
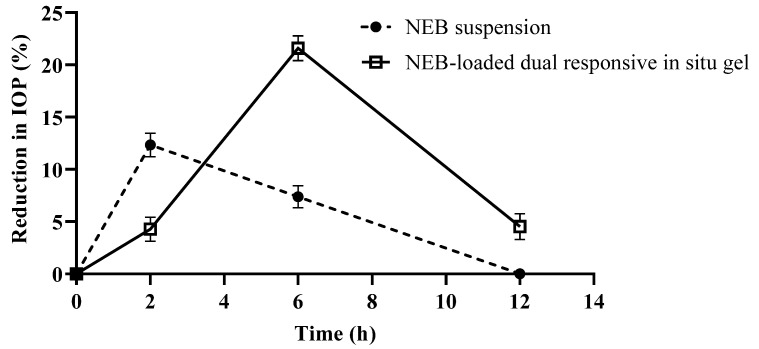
Percentage reduction in intra-ocular pressure [∆IOP (%)] versus time profiles obtained following ocular administration of an NEB suspension and optimized NEB-loaded dual-responsive in situ gel at a drug dose of 0.05 mg/kg in male New Zealand white rabbits (*n* = 6).

The percentage reduction in IOP [∆IOP(%)] versus time profiles of the NEB-loaded dual-responsive in situ gel and NEB suspension are presented in Figure 11. The pharmacodynamic data [∆IOP(%) versus time] of the two formulations was analysed using NCA to determine parameters such as area under the curve between ‘t = 0′ and ‘t = 12 h’ (AUC_0–12h_) and mean response time between ‘t = 0′ and ‘t = 12 h’ (MRT_0–12h_). The AUC_0–12h_ of the NEB-loaded dual-responsive in situ gel (137.04) was 1.85 fold higher (*P_cal_* < 0.0001) compared with that of the NEB suspension (74.21). A higher pharmacodynamic response was observed for the in situ gel compared to the NEB suspension. In addition, the MRT_0–12h_ of the in situ gel (6.1 h) was higher compared to the that of the NEB suspension (4.06 h). The NEB-loaded dual-responsive in situ gel could provide a sustained pharmacodynamic effect compared to the NEB suspension. These results are in line with the data obtained in the pharmacokinetic studies, which clearly indicated a higher and sustained concentration of NEB in the aqueous humor for NEB-loaded dual-responsive in situ gel compared to NEB suspension.

## 4. Conclusions

In the present study, NEB-loaded dual-responsive in situ gels containing a mixture of P407 + P188 as a thermo-responsive polymer and κCRG as an ion-responsive polymer was successfully developed and optimized using BBD. The optimized dual-responsive in situ gel exhibited the desired flow properties at room temperature while undergoing rapid sol-to-gel transition at physiological temperature in the presence of STF. The dual-responsive in situ gel was well tolerated with no signs of irritation/inflammation of the eye. The formulation showed good mucoadhesive characteristics. Ocular pharmacokinetic studies revealed that the optimized NEB-loaded dual-responsive in situ gel could enhance the ocular bioavailability with minimum systemic exposure compared to NEB suspensions. The pharmacodynamic studies established the efficacy of the NEB-loaded dual-responsive in situ gel in reducing the IOP compared to the NEB suspension. The results obtained in the current research showed that optimized NEB-loaded dual-responsive in situ gels can be a promising drug delivery system for the effective treatment of glaucoma.

## Figures and Tables

**Table 1:** Experimental design used in BBD for optimization of NEB-loaded dual-responsive in situ gels.

Factors	Levels Used		
	−1	0	+1
Independent Variables			
X_1_ = P407 concentration (% *w/v*)	18%	19%	20%
X_2_ = κCRG concentration (% *w/v*)	0.3%	0.4%	0.5%
X_3_ = P188 concentration (% *w/v*)	1%	3%	5%
**Dependent variables**	**Constraints**		
Y_1_ = Gelling temperature	In range of 33–35 °C		
Y_2_ = Solution state viscosity at 25 °C	Minimize		

**Table 2:** Design matrix of the 17 experimental runs generated by BBD and responses obtained from characterization of NEB-loaded dual-responsive in situ gels in terms of gelling temperature and solution state viscosity.

Run	Critical Factors			Response	
	P407 Concentration (X_1_, %*w/v*)	κCRG Concentration (X_2_, %*w/v*)	P188 Concentration (X_3_, %*w/v*)	Gelling Temperature (Y_1_, °C)	Sol State Viscosity (Y_2_, cP)
1	19	0.4	3	41	212
2	19	0.4	3	41	205
3	19	0.5	1	34	217
4	18	0.3	3	42	183
5	19	0.5	5	46	209
6	19	0.3	5	45	199
7	20	0.4	5	45	227
8	18	0.4	1	35	195
9	19	0.4	3	41	204
10	19	0.3	1	34	211
11	20	0.4	1	33	233
12	18	0.4	5	47	179
13	20	0.5	3	40	227
14	19	0.4	3	40	210
15	19	0.4	3	41	205
16	18	0.5	3	42	187
17	20	0.3	3	40	224

**Table 3:** Results obtained from ANOVA of BBD for optimization of gelling temperature and solution state viscosity of NEB-loaded dual-responsive in situ gels.

Source	Gelling Temperature (Y_1,_ °C)				Sol State Viscosity at 25 °C (Y_2,_ cP)			
	Sum of Squares	DF	F-Value	*p*-Value	Sum of Squares	DF	F-Value	*p*-Value
**Model**	289.07	9	214.12	<0.0001	3835.69	91	46.22	<0.0001
X_1_	8.00	1	53.33	0.0002	3486.13	1	378.05	<0.0001
X_2_	0.125	1	0.833	0.3917	66.13	1	7017	0.0316
X_3_	276.13	1	1840.83	<0.0001	220.50	1	23.91	0.0018
X_1_ X_2_	0.000	1	0.000	1.000	0.25	1	0.0271	0.8739
X_1_ X_3_	0.000	1	0.000	1.000	25	1	2.71	0.1436
X_2_ X_3_	0.250	1	1.67	0.2377	4.16	1	0.433	0.5312
X_1_^2^	0.213	1	1.42	0.2721	6.32	1	0.6852	0.4351
X_2_^2^	0.0026	1	0.0175	0.8984	2.21	1	0.240	0.6392
X_3_^2^	4.42	1	29.49	0.0010	26.84	1	2.91	0.1317
Residual	1.05	7			64.45	7		
Lack of fit	0.2500	3	0.4167	0.751	13.75	3	0.3609	0.7856
Pure error	0.800	4			50.80	4		
Total	290.12	16			3900.24	16		

**Table 4:** Loss tangent (tan δ) of optimized blank dual-responsive in situ gel and NEB-loaded dual-responsive in situ gel as a function of temperature (20 to 37 °C) in presence of STF and deionized (DI) water.

Formulation	Experimental Condition Used in Rheological Study		
	Only Temp Ramp	Temp Ramp in Presence of DI Water	Temp Ramp in Presence of STF
Blank dual-responsive in situ gel	tan δ > 1 in the range of 20–33 °C and tan δ = 1 at 34 °C	tan δ >> 1 in the range of 20–33 °C and tan δ = 1 at 34 °C	tan δ < 1 in the range of 20–30 °C and tan δ << 1 in the range of 30–37 °C
NEB-loaded dual-responsive in situ gel	20–30 tan δ > 1 At 32 °C tan δ = 1	tan δ >> 1 in the range of 20–32 °C and tan δ = 1 at 33 °C	tan δ < 1 in the range of 20–31 °C and tan δ << 1 in the range of 31–37 °C

Note—tan δ >> 1 indicates low storage modulus with no gelation (liquid state); tan δ > 1 indicates increased storage modulus with no gelation; tan δ = 1 indicates gelling point; tan δ < 1 indicates gelling with high storage modulus (gel with low viscosity) and tan δ << 1 indicates gelling with very high storage modulus (gel with high viscosity).

**Table 5:** Pharmacokinetic parameters of NEB in aqueous humor and plasma following ocular administration of an NEB suspension and optimized NEB-loaded dual-responsive in situ gel in male New Zealand white rabbits.

Biological Matrix	Parameters	Units	Treatments	
			NEB Suspension	NEB In Situ Gel
Aqueous humor	C_max_	ng/mL	28.2 ± 3.1	35.14 ± 2.25 *
	T_max_	h	2	4
	AUC_0–24_	ng/mL * h	189.0 ± 13.14	364.1 ± 16.76 ***
	AUC_0-∞_	ng/mL * h	194.9 ± 12.17	381.8 ± 18.32 ***
	MRT_0-∞_	h	6.12 ± 0.178	8.11 ± 0.12 ***
Plasma	C_max_	ng/mL	1.8 ± 0.01	0.6 ± 0.01 ***
	T_max_	h	0.5	1
	AUC_0–24_	ng/mL * h	20.2 ± 2.7	4.1 ± 0.2 ***
	AUC_0-∞_	ng/mL * h	33.2 ± 2.1	8.0 ± 0.43 ***
	MRT_0-∞_	h	25.8 ± 1.5	11.01 ± 0.6 ***

Each value represents the mean ± SD of four independent determinations (*n* = 4). * Statistically significant difference (*P_cal_* < 0.05) was observed when compared against the NEB suspension. *** Statistically significant difference (*P_cal_* < 0.0001) was observed when compared against the NEB suspension.

## Data Availability

Not applicable.
